# DECOLONIZING AGING RESEARCH AND PRACTICE

**DOI:** 10.1093/geroni/igae098.1467

**Published:** 2024-12-31

**Authors:** Lena Thompson, Steffi Kim, Jordan Lewis

**Affiliations:** Chair; Co-Chair; Discussant

## Abstract

The focus of this symposium, co-sponsored by the Indigenous Peoples and Aging Research Interest Group and Rural Aging Interest Group, is to highlight projects that apply Indigenous and/or non-Western perspectives to co-create research or programs for Indigenous people. While researchers and practitioners continue to work on closing the research implementation gap, a lack of involvement of Indigenous people in research and a lack of Indigenous-focused research leads to longer delays between culturally safe and effective research findings and program implementation. Furthermore, Indigenous communities have faced instances where institutions have been the primary beneficiaries of research conducted on Indigenous communities. Engaging Indigenous people and perspectives in the research process enhances the relevance and sustainability of research and allows a more equitable balance of benefits from research for all parties involved. The goal of this symposium is to present research studies that showcases methodologies and protocol outside of the Western academic perspective that focus on community-engaged, strengths-based approaches. Papers presented at the symposium will share methods used to enhance connectedness of Indigenous people to research and programs through the use of community-engaged approaches, storytelling, and qualitative data collection.

